# Non-pathogenic microflora of a spring water with regenerative properties

**DOI:** 10.3892/br.2015.507

**Published:** 2015-08-24

**Authors:** GIOVANNI NICOLETTI, MARTA CORBELLA, OMAR JABER, PIERO MARONE, DANIELE SCEVOLA, ANGELA FAGA

**Affiliations:** 1 Plastic and Reconstructive Surgery, Department of Clinical Surgical Diagnostic and Pediatric Sciences, University of Pavia, I-27100 Pavia, Italy; 2 Plastic and Reconstructive Surgery Unit, Salvatore Maugeri Research and Care Institute, I-27100 Pavia, Italy; 3 Advanced Technologies for Regenerative Medicine and Inductive Surgery Research Centre, University of Pavia, I-27100 Pavia, Italy; 4 Department of Infectious Diseases, San Matteo Research and Care Institute, I-27100 Pavia, Italy; 5 Department of Internal Medicine and Medical Therapeutics, University of Pavia, I-27100 Pavia, Italy

**Keywords:** spring water, Comano spring water, skin regeneration, microbiota, non-pathogenic bacterial species

## Abstract

The Comano spring water (Comano, Italy) has been demonstrated to improve skin regeneration, not only by increasing keratinocyte proliferation and migration, but also by modulating the regenerated collagen and elastic fibers in the dermis. However, such biological properties may not be entirely explained by its mineral composition only. As the non-pathogenic bacterial populations have demonstrated an active role in different biological processes, the potential presence of non-pathogenic bacterial species within the Comano spring water was investigated in order to identify any possible correlation between these bacterial populations and the demonstrated biological properties of this water. The water was collected at the spring using an aseptic procedure and multiple cultures were carried out. A total of 9 different strains were isolated, which were *Aeromonas hydrophila*, *Brevundimonas vesicularis*, *Chromobacterium violaceum*, *Citrobacter youngae*, *Empedobacter brevis*, *Pantoea agglomerans*, *Pseudomonas putida*, *Pseudomonas stutzeri* and *Streptococcus mitis*. All the isolated bacterial strains, although showing a rare potential virulence, demonstrated peculiar and favorable metabolic attitudes in controlling environmental pollution. The therapeutical effects of certain spring waters are currently being proven as correlated not only to their peculiar mineral composition, but also to the complex activity of their resident non-pathogenic bacterial populations. Although the present study provided only preliminary data, some of the non-pathogenic bacterial populations that were identified in the Comano spring water are likely to produce molecular mediators with a role in the wound healing process that, thus far, remain unknown. Numerous other unknown bacterial species, comprehensively termed DNA-rich ‘dark matter’, are likely to contribute to the Comano water regenerative properties as well. Therefore, the non-pathogenic bacterial populations of the Comano spring water are possibly credited for its demonstrated regenerative properties.

## Introduction

The benefits of spring waters in the treatment of actual pathologies and/or in re-establishing the physiological wellness of different organs and systems have been demonstrated since the most ancient times, and specific indications have been historically attributed to each spring.

However, the molecular mechanisms and the various interactions responsible for the anti-inflammatory and regenerative properties of spring waters remain largely unknown and require investigation.

Our previous study demonstrated that an Italian spring water (Comano-Trentino) can improve skin regeneration in an animal experimental model, not only by increasing keratinocyte proliferation and migration, but also by modulating the regenerated collagen and elastic fibers in the dermis (1).

However, such biological properties may not be entirely explained by the mineral composition only.

As the non-pathogenic bacterial populations have demonstrated an active role in different biological processes, the potential presence of non-pathogenic bacterial species within the Comano spring water have been investigated in order to identify any possible correlation between these bacterial populations and the demonstrated biological properties of this water.

## Materials and methods

### 

#### General

**Figure 1.**
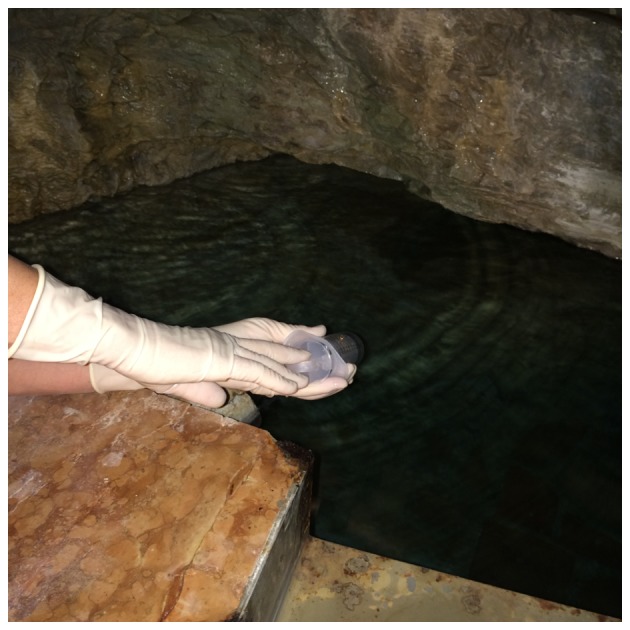
Water collection at the spring with aseptic procedure using sterile gloves and a sterile 50 ml syringe.

The Comano spring water was collected at the spring with an aseptic procedure (Fig. 1) in January, June and October 2014. A single operator wearing sterile surgical gloves collected 3,000 ml of water each time with a sterile 50-ml syringe. The samples were poured into 3 sterile one-liter containers for microbiological analysis and stored at 4°C.

#### Sample processing, isolation and identification of bacteria

Samples were transported to the Laboratory of Bacteriology, Microbiology and Virology Department, San Matteo Hospital Foundation, Research and Care Institute (Pavia, Italy), at 4°C and processed rapidly following collection.

Two pairs of BD BACTEC™ culture aerobic/anaerobic vials were inoculated each with 10 ml water. Subsequently, the samples were incubated in a BACTEC™ 9240 automated blood culture system (BD Biosciences, Sparks, MD, USA), according to the manufacturer's instructions, for 7 days.

Six 0.20-µm pore cellulose nitrate membranes (Nalgene 0.2 Analytical filter Unit; Thermo Fisher Scientific, Inc., Waltham, MA, USA) were used to filter 100 ml of water each. Five membranes were subsequently placed on a different plating medium (blood, chocolate, McConkey, mannitol salt or Sabouraud dextrose agars) and incubated in aerobic conditions at 37°C for 3 days. One membrane was placed on Schaedler blood agar medium and incubated in anaerobic conditions at 37°C for 6 days.

Similarly, a 1,000-ml water sample was filtered through a 0.20-µm pore cellulose nitrate membrane and processed according to the guidelines for *Legionella* detection in water (2). In particular, buffered charcoal yeast extract and glycine, vancomycin, polymyxin B and cycloheximide plates were inoculated and incubated at 37°C with 5% CO_2_ for 14 days.

Isolated organisms were biochemically identified with API NE (BioMerieux SA, Marcy l'Etoile, France) or using the Phoenix 100™ (BD Biosciences) automated system.

## Results

### 

#### Positive identification of cultures

BD BACTEC™ culture aerobic/anaerobic vials became positive after 3 days.

#### Biochemical identification of the bacterial cultures

The biochemical identification of the cultured bacteria provided different results at different times. The Phoenix 100™ (BD Biosciences) automated system identified *Citrobacter youngae* and *Pantoea agglomerans* in January 2014, *Pseudomonas stutzeri* and *Streptococcus mitis* in June 2014 and no colonies in October 2014. The microorganisms isolated with API NE (BioMerieux sa) were *Aeromonas hydrophila* (CB 6 UFC/100 ml), *Chromobacterium violaceum* (CB 2 UFC/100 ml) and *Empedobacter brevis* (CB 3 UFC/100 ml) in January 2014, *Brevundimonas vesicularis* (CB 7 UFC/100 ml) and *Pseudomonas putida* (CB 4 UFC/100 ml) in June 2014, and *Aeromonas hydrophila* (CB 3 UFC/100 ml) and *Pseudomonas putida* (CB 8 UFC/100 ml) in October 2014.

The isolates at different times per identification system are summarized in Table I. A classification of the isolates is provided in Table II.

## Discussion

The Comano spring water is a hypotonic, bicarbonate-calcium-magnesium mineral water that is rich in fluoride, and has a neutral pH and a low-buffer capacity. The ECOOPERA S.C. laboratory (Gardolo, TN, Italy; ACCREDIA certified no. 0252) regularly certifies this water as bacteriologically pure, the latter definition meaning that it does not contain pathogenic microorganisms nor microorganisms indicating fecal or other contamination (3).

A total of 9 different strains were isolated from the Comano spring water: *Aeromonas hydrophila*, *Brevundimonas vesicularis*, *Chromobacterium violaceum*, *Citrobacter youngae*, *Empedobacter brevis*, *Pantoea agglomerans*, *Pseudomonas putida*, *Pseudomonas stutzeri* and *Streptococcus mitis*.

*Aeromonas hydrophila* is the most prominent of the 6 species of *Aeromonas* (4). It is a heterotrophic Gram-negative bacterium mainly identified in areas with a warm climate. This bacterium can be found in fresh or brackish water and can survive in aerobic and anaerobic environments. *Aeromonas hydrophila* represents a constant component of the microbiota in fresh reservoirs where, together with other microorganisms, it acts as a natural biofilter and promotes water self-purification. It is present in normal microflora of hydrobionts inhabiting fresh reservoirs (5). Recently, *Aeromonas hydrophila* was isolated in samples from Moroccan Atlantic Ocean water (6), where antifungal and antibacterial activity was demonstrated.

*Brevundimonas vesicularis* is an environmental Gram-negative bacillum that has demonstrated the capacity to degrade sulfonated naphthalene-formaldehyde condensate compounds isolated from textile industry-activated sludge wastewater (7).

*Chromobacterium violaceum* is a facultative anaerobe Gram-negative bacterium, considered to be non-pathogenic for humans. This bacterium appears to control microbial infection and decrease the risks of resistance development (8). Violacein, the pigment of *Chromobacterium violaceum*, has potential medical applications as a drug with different properties: Antitrypanocidal, antileishmaniosis, antimycobacterial, antimalaric, anti-ulcerogenic, anticancer and antioxidant (9). *Chromobacterium violaceum* was also isolated in samples from Moroccan Atlantic Ocean water (6).

The *Citrobacter youngae* species are straight, facultative anaerobic Gram-negative bacilli that are commonly found in water, soil, food and in the intestinal tracts of animals and humans. Certain strains belonging to the genus *Citrobacter* have been reported to produce chitin/chitosan-like bioflocculants from acetate (10). Furthermore, degradation of the lipopolysaccharide of *Citrobacter youngae* releases polysaccharides with structure rarely identified in bacteria (11).

*Empedobacter brevis*, a Gram-negative aerobe also known as *Flavobacterium breve*, is the only member of the *Empedobacter* genus. It produces an enzyme that catalyzes the peptide-forming reaction producing l-alanyl-L-glutamine, a dipeptide of significant industrial interest by virtue of its widespread use in infusion therapy (12).

*Pantoea agglomerans* is a Gram-negative bacterium that belongs to the family *Enterobacteriaceae*. It is commonly isolated from plant surfaces, seeds, fruit (such as mandarin oranges) and animal or human feces. *Pantoea agglomerans* is primarily a plant epiphyte commonly found in diverse ecological niches, including aquatic environments, soil or sediments. Several strains of *Pantoea agglomerans* are sold as commercial biological control agents against the fire blight pathogen on apple and pear trees (13). The primary mode of action is competitive exclusion, which involves the occupation of sites otherwise colonized by the pathogens; however, according to the literature, certain strains may also contribute with the production of different antibiotic-like substances (herbicolins, pantocins, putative phenazine and other unknown compounds) (14). This bacterium was also isolated in samples from Moroccan Atlantic Ocean water (6).

*Pseudomonas putida* is a Gram-negative aerobic saprotrophic soil bacterium. As it is able to degrade organic solvents, such as toluene, and also convert styrene oil to biodegradable plastic polyhydroxyalkanoates, it may be used to degrade the polystyrene foam that was thought to be non-biodegradable (15). Furthermore, one of its engineered strains proved to be useful for *in situ* bioremediation of soils co-contaminated with organophosphorus and pyrethroid pesticides. In turn, *Pseudomonas putida* induces plant growth and protects the plants from pathogens. Τherefore, researchers use it in bioengineering research to develop biopesticides and to the improve plant health (16).

*Pseudomonas stutzeri*, an almost universal Gram-negative ammonia-oxidizing bacterium, exhibits abilities in efficient heterotrophic nitrification and aerobic denitrification (17), and in organophosphorus pesticides degradation (18). Therefore, it is considered a suitable candidate to simultaneously remove nitrogen and phosphate in wastewater treatment. Similar to *Brevundimonas vesicularis*, it also demonstrated the capacity to degrade sulfonated naphthalene-formaldehyde condensate compounds isolated from textile industry-activated sludge wastewater (6).

*Streptococcus mitis* is a Gram-positive facultative anaerobe coccus that is an abundant human oral commensal and is reported as a potentially useful vector for mucosal vaccination (19).

While in the past microorganisms were recognized just as enemies of the human body, the trend has currently changed as the position of microbiota has recently undergone a turning point in the contemporary vision of medicine. Helminths, saprophytic mycobacteria, *bifidobacteria* and *lactobacilli* cause little, if any, harm and have been a part of human microecology for millenia. Deficient exposure to these may even explain the contemporary increase of immune disorders in a modern, highly sanitized society (20).

The use of probiotics, prebiotics, helminths or microbe-derived immunoregulatory substances has become a novel and valuable approach to disease prevention (20), and emerging clinical studies indicate that the supplementation and/or fecal microbial transplant (21) can improve bowel health and brain functions (22,23).

The skin microbiota is constituted by bacteria and fungi with typical counts of 10^2^−10^7^ cells/cm^2^ in a diverse topography reflecting their different niches (24,25).

Normal skin microorganisms are classified as either resident (i.e. adhering predominantly to the skin and annexes, maintaining viability and reproducibility) or transient (i.e. deposited but not adhering to the skin surface, with little nor sustained growth and reproduction). The skin ecosystem is a complex environment, extending to sub-epidermal compartments (26), tending to withstand pathogen colonization. Stability is maintained by interactions among different microbial species and the host. The topical use of probiotics has been reported to have a direct effect on the site of application, as natural defence mechanisms are induced by the competition with pathogens for nutrients, by the modulation of mucosal immune functions and by the production of antimicrobial metabolites (27–29).

Furthermore, epidermal keratinocytes have been demonstrated to produce antimicrobial peptides (30–32).

A correlation between the well-known beneficial effects on the skin and the resident non-pathogen bacterial populations was demonstrated in certain spring waters.

From the culture of *Aquaphilus dolomiae*, a non-spore forming bacterium belonging to the Neisseriaceae family isolated from Avène thermal Water (France), an organic substance, I-modulia, was obtained that is able to regulate keratinocyte inflammatory and lymphocyte immune responses (33–36).

Similarly (37,38), the topical administration of a lysate of *Vitreoscilla filiformis*, a Gram-negative aerobic bacterium belonging to the Neisseriaceae family found in LaRoche-Posay thermal water (France), has been demonstrated to benefit the local skin immunity, possibly by the activation of cutaneous regulatory T cells (39,40).

Our previous study on the properties of the Comano water on experimental fresh wounds in an animal model demonstrated a significant increase in the overall cell proliferation and a corresponding reduction of the local inflammatory response (1).

Such effects may not be entirely explained by the mineral composition only, but may be correlated to the antifungal and antimicrobial properties of some of the bacterial isolates as well (40).

Although showing a rare potential virulence (41–50), all the isolated bacterial strains demonstrate peculiar and favorable metabolic attitudes in controlling environmental pollution.

Skin regeneration is a complex process involving the close and coordinated interaction of different cell strains, as keratinocytes, fibroblasts and immune-system cells in the extracellular matrix environment.

The modulation of the wound proliferative process is likely to be influenced by the local microbiota as well, and this hypothesis has not been investigated previously. Therefore, it appears reasonable to conceive this study with the aim to provide a more comprehensive understanding of the regenerative properties of the Comano spring water.

Although the present study provided only preliminary data, some of the non-pathogenic bacterial populations that were identified in the Comano spring water are likely to produce molecular mediators with a role in the wound healing process that, thus far, remain unknown. Numerous other unknown bacterial species, comprehensively termed DNA-rich ‘dark matter’, are likely to contribute to the Comano water regenerative properties as well.

In conclusion, the therapeutical effects of certain spring waters are currently being proven as correlated not only to their peculiar mineral composition, but also to the complex activity of their resident, non-pathogenic bacterial populations (33–40).

Therefore, the non-pathogenic bacterial populations of the Comano spring water are likely to be credited for its demonstrated regenerative properties (1).

Such evidence may direct the introduction of novel research opportunities for regeneration to the role of microbiota.

## Figures and Tables

**Table I.** Isolates at different times per identification system.

	Identification system	
	
Dates	Phoenix 100™	API NE
January 2014	*Citrobacter youngae*	*Aeromonas hydrophila* (CB 6 UFC/100 ml)
	*Pantoea agglomerans*	*Chromobacterium violaceum* (CB 2 UFC/100 ml)
		*Empedobacter brevis* (CB 3 UFC/100 ml)
June 2014	*Pseudomonas stutzeri*	*Brevundimonas vesicularis* (CB 7 UFC/100 ml)
	*Streptococcus mitis*	*Pseudomonas putida* (CB 4 UFC/100 ml)
October 2014	Negative culture	*Aeromonas hydrophila* (CB 3 UFC/100 ml)
		*Pseudomonas putida* (CB 8 UFC/100 ml)

**Table II.** Classification of isolates.

Isolates	Family	Genus	Species
*Aeromonas hydrophila*	Aeromonadaceae	Aeromonas	*H. hydrophila*
*Brevundimonas vesicularis*	Caulobacteriaceae	Brevundimonas	*B. vesicularis*
*Chromobacterium violaceum*	Neisseriaceae	Chromobacterium	*C. violaceum*
*Citrobacter youngae*	Enterobacteriaceae	Citrobacter	*C. youngae*
*Empedobacter brevis*	Flavobacteriaceae	Empedobacter	*E. brevis*
*Pantoea agglomerans*	Enterobacteriaceae	Pantoea	*P. agglomerans*
*Pseudomonas putida*	Pseudomonadaceae	Pseudomonas	*P. putida*
*Pseudomonas stutzeri*	Pseudomonadaceae	Pseudomonas	*P. stutzeri*
*Streptococcus mitis*	Streptococcaceae	Streptococcus	*S. mitis*
